# Preliminary Design of a Model-Free Synthetic Sensor for Aerodynamic Angle Estimation for Commercial Aviation †

**DOI:** 10.3390/s19235133

**Published:** 2019-11-23

**Authors:** Angelo Lerro, Alberto Brandl, Manuela Battipede, Piero Gili

**Affiliations:** Department of Mechanical and Aerospace Engineering, Politecnico di Torino, C.so Duca degli Abruzzi 24, 10129 Torino, Italy; alberto.brandl@polito.it (A.B.); manuela.battipede@polito.it (M.B.); piero.gili@polito.it (P.G.)

**Keywords:** air data system, flight dynamics, state observer, synthetic sensor, virtual sensor, analytical redundancy, avionics, neural network

## Abstract

Heterogeneity of the small aircraft category (e.g., small air transport (SAT), urban air mobility (UAM), unmanned aircraft system (UAS)), modern avionic solution (e.g., fly-by-wire (FBW)) and reduced aircraft (A/C) size require more compact, integrated, digital and modular air data system (ADS) able to measure data from the external environment. The MIDAS project, funded in the frame of the Clean Sky 2 program, aims to satisfy those recent requirements with an ADS certified for commercial applications. The main pillar lays on a smart fusion between COTS solutions and analytical sensors (patented technology) for the identification of the aerodynamic angles. The identification involves both flight dynamic relationships and data-driven state observer(s) based on neural techniques, which are deterministic once the training is completed. As this project will bring analytical sensors on board of civil aircraft as part of a redundant system for the very first time, design activities documented in this work have a particular focus on airworthiness certification aspects. At this maturity level, simulated data are used, real flight test data will be used in the next stages. Data collection is described both for the training and test aspects. Training maneuvers are defined aiming to excite all dynamic modes, whereas test maneuvers are collected aiming to validate results independently from the training set and all autopilot configurations. Results demonstrate that an alternate solution is possible enabling significant savings in terms of computational effort and lines of codes but they show, at the same time, that a better training strategy may be beneficial to cope with the new neural network architecture.

## 1. Introduction

Air data systems (ADSs) are adopted on air vehicles to measure a set of data from the external environment. Generally speaking, a simplex ADS is made up of external (i.e., installed externally on the aircraft (A/C) fuselage) probes and vanes able to measure a full set of air data:

- local static pressure, $P_{s}$;
- local total pressure, $P_{0}$;
- local air temperature (static, outside air temperature (OAT), or total, total air temperature (TAT));
- local angle of attack (AoA), α;
- local angle of sideslip (AoS), β.

The ADS probes/vanes are connected (or integrated) with a corresponding measuring module (air data modules (ADMs)) encompassing suitable transducers able to convert the measure into analog (or digital) signals. If those ADM are all embedded in a single box, it usually refers to a central processor unit (air data computer, (ADC)). The ADC is able to provide pilots, or flight control computers, FCCs, with the more relevant air data information necessary for piloting, navigation and control purposes. In recent years, the air data algorithms are often implemented into the FCC and the ADC is removed. In both cases, the air data functionalities shall be able to calculate the following parameters:

- Pressure altitude
- Pressure altitude, baro-corrected (Kollsman)
- Vertical speed
- Calibrated airspeed (CAS)
- Equivalent airspeed (EAS)
- True airspeed (TAS) (only if OAT or TAT is available)
- Mach number
- Air temperature, $T_{\infty }$, (only if OAT or TAT is available)
- AoA
- AoS

Generally speaking, for each element of the previous list except for AoA and AoS, the standard AS8002A [1] sets operative performance and environmental requirements. For aerodynamic angles, AoA and AoS, there are not clear and well-defined performance requirements. Even though the AoA measurements shall satisfy the standard AS403A [2], this standard sets prescriptions only for stall protection purposes and not for the entire range of flyable angle of attack. There is no standard applicable for AoS. In fact, when AoA and AoS are required to the ADS, the functional requirements are usually derived from other functionalities as described later in Section 3.2.

According to the function allocated to each air data measured, the A/C integrator with failure hazard analysis (FHA) will classify the criticality of the loss of each one of the air data measured or calculated. There are some parameters, e.g., the CAS, whose loss is always classified as catastrophic and therefore the corresponding air data become safety-critical. The ADS, therefore, is one of the safety-critical systems on board that should be redundant in order to meet the A/C category safety requirement: for example, triplex solution is the common standard in commercial aviation.

With modern technologies, recently the ADS moved towards digital solutions for a better integration with modern digital avionics. Fly-by-wire (FBW) paradigm is successfully applied to large aircraft and therefore a more electrical aircraft is a technological transition necessary to achieve the goals defined by the European Community, EC, within the FlightPath 2050 [3]. The aeronautical industry has launched several programs to cope with the fly-by-wire challenge, even for those systems that seemed less involved in this revolution, such as the ADS. In fact, in order to overcome drawbacks to connect probes and vanes to ADMs pneumatically and then each ADM to the flight control system (FCS), many recent large FBW A/C are equipped with integrated probes that embed transducers within the probe (or vane) itself [4].

The same approach is shared with those air vehicles of reduced size, e.g., small aircraft transport (SAT) category, unmanned aircraft system (UAS), and urban air mobility vehicles (UAM), where the FBW is necessary for a more-integrated system. SAT aircraft is a crucial segment for travelers in Europe because it is the only means of transportation that has those characteristics able to fill a gap that could not be done in another way. Short-range flights, local communities’ connections, door-to-door within four hours [3] are only a few examples that highlight the importance of the SAT segment in the European infrastructures. Efforts spent worldwide by governments and aviation safety agencies to regulate the air work of UAS and UAM vehicles over populated areas is a clear evidence that what has been studied by many companies could take off in the next years. As far as ADS is concerned, SAT, UAS and UAM categories have common drawbacks, e.g., heterogeneity of ADS requirements due to high range of A/C mission (altitude, speed, etc.) and need to optimize ADS’s line replaceable units (LRUs) installation on board in terms of space and weight due to reduced fuselage size (if compared with civil aircraft). Each aircraft will have its own dedicated probes/vanes to satisfy safety and performance requirements. This particular aspect would suggest to rely on a solid core in order to have a multi-platform ADS with interchangeable external probes.

ADS’s safety is another crucial aspect to be taken into account. In order to increase the reliability of ADS, a physical redundancy is applied. Moreover, there are other requirements from airworthiness authorities that should be taken into account, e.g., those related to the bird strike, that set some constraints on the fuselage installations. Analytical redundancy [5,6,7] is a concept used more and more frequently in recent years because the avionic background is mature to welcome such innovations. This approach enables the replacement of physical sensors (used for redundancy) with analytical ones for aerodynamic angles [8,9,10] and airspeed [11] with several benefits in terms of weight, power consumption, reliability, maintainability, and emissions.

The advent of distributed avionics, e.g., ARINC 664 networks on Airbus A380, A400M, A350 and Boeing 787, has been seen as a significant booster for a better exploitation of onboard data to be used, for instance, by other subsystems for redundancy purposes.

Within this scenario, innovative ADS for FBW applications as part of a redundant ADS is introduced with the MIDAS project funded in the SAT category of Clean Sky 2 programme [12].

The MIDAS ADS will be driven by an integrative, modular and digital approach. Following the market trend and EC guidelines, the project outcome will be a fully-integrated probe (air data probes integrated with electronics) with digital outputs that can be interfaced with modern avionic bus onboard FBW A/C and several classes of external Pitot tubes and TAT probes.

The main innovation behind the MIDAS ADS lays on a patented technology [13], named smart-air data system, attitude and heading reference system (ADAHRS), firstly by Politecnico di Torino and later under AeroSmart S.r.l. [14] responsibility. This solution, basically a state observer obtained with a data-driven methodology, is able to estimate AoA and AoS with analytical sensors [15,16,17] exploiting A/C flight dynamic equations and onboard data fusion.

The MIDAS equipment will be qualified (both for hardware [18] and environmental [19] aspects) and, therefore, even the virtual sensors. This aspect makes the MIDAS project a fundamental milestone in the certification process of analytical estimators for civil applications. Therefore, the main outcome of this project is to provide a qualifiable electronics able to be integrated into modern avionic bus (e.g., ARINC 664 network) that can be interfaced with several COTS probes (Pitot/static and TAT probes already certified by their own manufacturers).

This work deals with preliminary design activities accomplished to define analytical sensors (or virtual ones) for AoA and AoS estimation exploiting simulated flight data. The final design will deliver a qualifiable software module to be integrated on a demo board with AoA and AoS estimators guaranteeing at least the same performance of state-of-the-art sensors. Mainly for certification reasons, moving from previous and consolidated practice, a more efficient (in terms of computational cost and number of lines of code) virtual sensor will be designed for civil operative scenarios. Side findings emerge from the present work and they will affect the future steps of the MIDAS projects. The paper aims to highlight the original elements emerged during the present work and, therefore, neural network architecture optimization will not be discussed here because similar to previous works [20,21].

Considering the current project’s development stage, a complete and detailed reliability analysis cannot be conducted yet. For the same reason, the technology has been compared with simulated data. Even though the comparison with classical sensors is not described in this work, some brief considerations on expected performances will be given in Section 3.1.

This paper begins with an overview of the MIDAS ADS and comparison between the state-of-the-art in Section 2. Section 3 describes the preliminary design of the MIDAS ADS solution with details about the virtual sensor design (Section 3.1) and the certification aspects (Section 3.2) that can be condensed in well-established target performance. After the reference A/C is introduced in Section 4, the training strategy adopted in this work is presented in Section 5. Section 6 collects preliminary results obtained using only test maneuvers that are completely independent of the training pattern. The paper concludes with Section 7.

## 2. Approach

**Figure 1:**
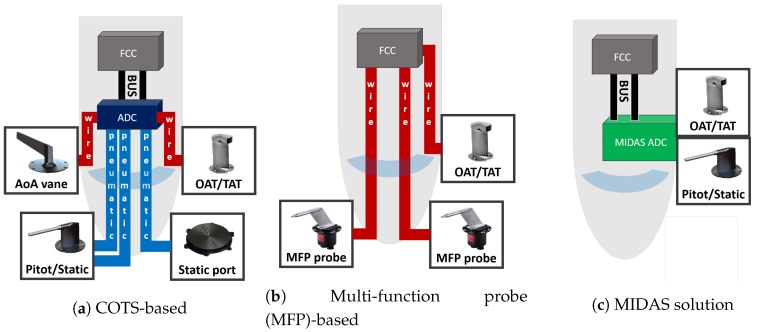
Schematic view of three realistic simplex air data system (ADS) architectures able to provide a complete set of air data.

Possible certifiable architectural solutions are made up of certified probes, vanes, and air data units (ADUs) and/or ADC. The adjective *certifiable* refers to a system suitable for commercial flights, and not only for experimental purposes, according to applicable airworthiness regulations [22]. The Figure 1 shows schematically realistic solutions. A brief description of the three kinds of realistic architectures will be given in this section.

### 2.1. ADS Based on COTS

Pressure probes and static ports are pneumatically connected to a central air data computer whereas the flow angle vanes and the TAT sensor are usually electronically connected to the ADC. Traditionally, the ADC contains several ADUs, essentially pressure transducers, which convert pressures into digital signals. Usually, the ADC has computational capabilities that are used to calibrate measured air data (e.g., local to freestream correction) and to calculate other air data parameters (e.g., mach number, pressure altitude, etc.). Today, the ADC has commonly been replaced by several ADM located near, or integrated into, the reference probe/vane with dedicated transducers. ADM’s main function is to convert data measured into digital ones.

The main drawbacks of ADS based on COTS LRUs are related to weight, encumbrance limitations and power consumption requirements (mainly for de-icing purposes).

### 2.2. ADS Based on Multi-Function Probes

Another solution for ADS is based on multi-function probes (MFPs). Generally speaking, MFP are digital LRUs able to embed Pitot tube, static port and one flow angle with an ADM. By means of the combination of two MFPs and two TAT sensors, it is possible to define a duplex ADS architecture (but simplex for AoS), while using three MFPs and three TAT sensors it is possible to define a triplex ADS architecture (but duplex for AoS). The MFPs and the TAT can be digitally connected to cockpit display systems and, in the case of FBW aircraft, it can be sent to the FCC. As only a few ADC functionalities are carried out at ADM level, the main ADC’s work is done at FCC level. The main drawbacks of the MFP-based solution are related to high costs and limited availability from very few companies all over the world.

### 2.3. ADS Based on MIDAS Solution

The focus of the MIDAS Project is shifted from probe/vane to flight mechanics using machine learning techniques and flight data already available onboard through a patented technology [13]. The MIDAS’s approach will introduce synthetic aerodynamic angle sensors more suitable for a dynamic aeronautical segment as the SAT, UAS or UAM. The MIDAS ambition is to provide an ADS solution that joins all benefits of flyable ADS architectures (Section 2.1 and Section 2.2) with practical elimination of their drawbacks. In particular, the main benefits will be:

- low weight and space;
- reduced power consumption with consequent lower emissions;
- no more than two external probes (COTS Pitot/static and TAT (or OAT) probes);
- easy to be installed on the fuselage: no requirements about front or nose installation;
- flexibility to be interfaced with a wide range of probes in order to cope with heterogeneity of ADS requirements from A/C of the CS23 category;
- possible ITAR free ADS system, thanks to the availability of necessary probes available from several suppliers all over the world.

## 3. MIDAS Technological Solution

**Figure 2:**
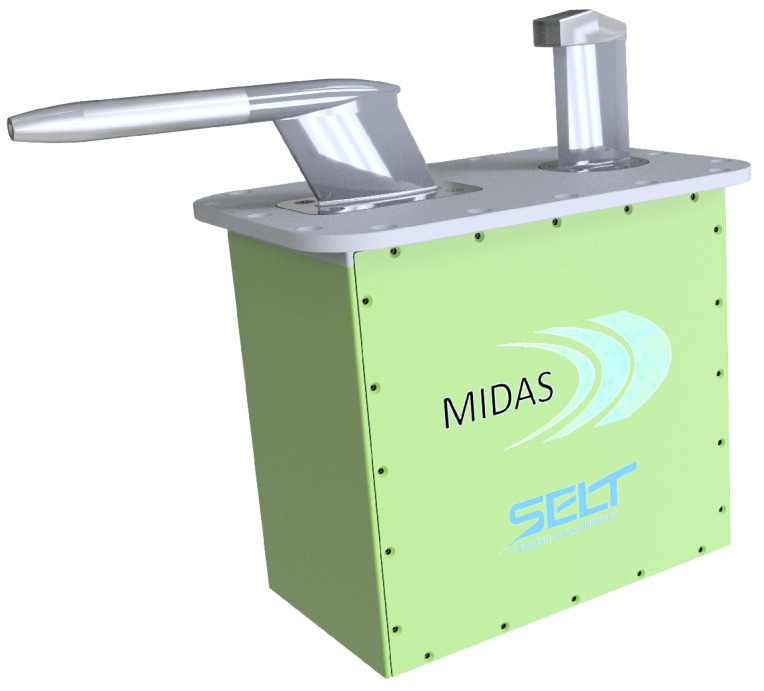
Preliminary MIDAS configuration. Courtesy of SELT A&D [23].

Today, two distinct probes (certified) to measure pressure and temperature for flyable ADS are needed. There are few attempts worldwide to integrate both measures into a single probe, but there are no certified products so far. Therefore, MIDAS approach is to integrate the COTS probes (Pitot-static and temperature probes) as close as possible, as in Figure 2, with dedicated electronics (MIDAS ADC) in order to achieve objectives defined later in Section 3.2.

Therefore, the MIDAS air data system will be made up of:

- ADC—electronic unit (embedding estimator(s) for AoA and AoS);
- pitot-static probe;
- TAT probe.

The MIDAS project expects a tandem solution of the external probes according to preliminary considerations, but a more detailed aerodynamic study will be performed to find the optimal configuration.

### 3.1. Virtual Sensors

As stated before, the MIDAS project’s outcome will provide a single LRU embedding virtual sensor (or sensors), dedicated to AoA and AoS estimation, based on a patented technology at TRL6 [24,25]. These virtual sensors are essentially state observers for which the A/C flight dynamic model is replaced by a model based on neural networks.

Exploiting the paradigm of the FBW aircraft, the air data probe (ADP) will receive, as input, consolidated data from other A/C equipment to be fused with measured ones ($P_{s}$, $P_{t}$ and $T_{\infty }$) in order to estimate AoA and AoS with high reliability.

The input and the output signals will be transmitted through the avionic bus of the A/C. As stated in the introduction, being the ADS a safety-critical system, the proposed solution must be considered valid also for redundant architectures in a hybrid framework, merging classical sensors and the MIDAS technology. The A/C integrator will be in charge to design a redundant ADS able to meet the applicable safety requirements with the best compromise merging COTS and synthetic sensors. The detailed reliability analysis of the MIDAS technology is non-trivial because it involves reliability analysis of other proprietary A/C systems (e.g., the FCC). Although some information can be deduced from previous works [15], this topic will be extensively dealt with in a future step of this project.

**Figure 3:**
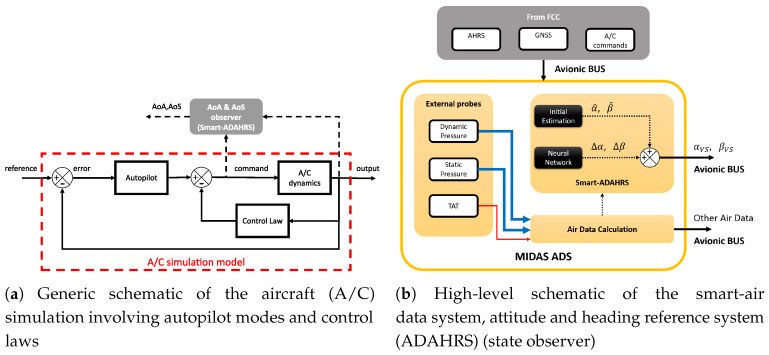
Generic schematics of A/C simulation and angle-of-attack (AoA)/angle-of-sideslip (AoS) estimators.

The virtual sensors proposed in the MIDAS project rely on the use of A/C data from attitude and heading reference system, primary surface commands/deflections and Global Navigation Satellite System (GNSS) (Figure 3b).

Topologically speaking, it consists of a biased linear combination of non-linear activation functions, each activation function is driven by a biased linear combination of the output of the preceding nodes. Although the multilayer perceptron (MLP) can be described from several points of view, in this case, the best description is that it can represent a non-linear map between the input and the target. The point is to find those weights of the network such that the output fits the desired map. The validity of the approach is mathematically proven using the universal approximation theorem (UAT). In fact, it is proven that any continuous function of *n* real variables, with support in the unit hypercube, can be uniformly approximated by finite superposition of a fixed, univariate function that is discriminatory [26].

The Smart-ADAHRS project deals with a very straightforward model, suitable for real-time and cost effective innovative avionic systems. Consider valid the following assumption on AoA and AoS:

(1) $$\begin{array}{cc} \alpha_{\mathrm{VS}} & =\hat{\alpha }+\Delta \alpha \; \end{array}$$

(2) $$\begin{array}{cc} \beta_{\mathrm{VS}} & =\hat{\beta }+\Delta \beta \end{array}$$

where $\hat{\alpha }$ and $\hat{\beta }$ are initial estimation obtained with flight mechanics equations whereas Δα and Δβ are the differences between the linear estimations and the true nonlinear values. According to a patented procedure [13], $\hat{\alpha }$ and $\hat{\beta }$ is augmented with the evaluation of Δα and Δβ based on two MLPs, which process measurements obtained with non-protruding sensors (except for the Pitot tube and TAT).

$\hat{\alpha }$ and $\hat{\beta }$ can be evaluated as follows:

(3) $$\begin{array}{cc} \hat{\alpha } & =\theta \;-\gamma \; \end{array}$$

(4) $$\begin{array}{cc} \hat{\beta } & =K\frac{n_{y}}{q_{c}} \end{array}$$

where θ stands for the pitch angle, γ for the flight path angle, $n_{y}$ is the proper acceleration as measured by the accelerometer along the $Y_{B}$ axis and $q_{c}$ is the impact pressure. *K* is an A/C constant derived from flight mechanic considerations (the order of magnitude for this category is 10^3^ kg m^−2^).

The patented approach can bring to a neural network with limited output(s) that is crucial aspect when dealing with certification authorities. Generally speaking, using real or simulated flight data the value ranges of Δα and Δβ can be identified. Our approach is to apply the limited output only once the neural network is trained. This strategy allows to train the neural network without any limitations and to bound the estimated AoA and AoS to avoid any overshoot or spike values during the operative life.

Mathematical demonstrations exist [26,27,28,29,30,31] about the MLP performing as a universal approximator. During the training procedure, the weights of the linear combinations are estimated solving the non-convex problem of the error function optimization. Different heuristic rules exist and the Levenberg-Marquard, LM, algorithm is used in this work. The complete input vector needed by Smart-ADAHRS includes data from the GPS (providing $V_{down}$), the ADS (providing TAS) and the attitude and heading reference system (AHRS) (providing angular rates, Euler angles and linear accelerations), as can be seen in Figure 3b. Figure 3a shows a generic flight simulator model with autopilot and control laws in the loop. The Smart-ADAHRS is basically a data-based state observer exploiting neural functionalities and flight mechanic equations as described in Figure 3b.

Previous research [32] showed that analytic evaluation is indeed feasible for the evaluation of AoA and AoS, thanks to on board available data and with dedicated virtual sensors, one trained ad hoc for AoA and another for AoS. With the present application, where aircraft complexity is higher (for the auotpilot and control law presence itself), the scenario changes. Even though the presence of autopilot modes and control laws, as well known, affect the A/C dynamic behavior, they do not influence generic state observers if they are fed with current output and control surfaces, as showed in Figure 3a.

Another important aspect is related to real operating scenario: a common virtual sensor able to estimate at the same time both AoA and AoS (essentially a single neural network with double output) could be beneficial in terms of required computational time and for ceritification aspects as mentioned before. In order to provide a realistic comparison, three feed-forward predictors are designed, (i) virtual, analytical or synthetic sensor (VS)-AoA, (ii) VS-AoS and (iii) VS-A&S, with the same architecture, the same input vector and the same training path (as shown in Table 1).

The virtual sensors considered in this work have the following characteristics:

- feed-forward neural network;
- one hidden layer with 24 neurons;
- neurons with sigmoidal activation functions;
- one output layer with a single (or double for the VS-A&S) linear neuron;
- limited output during the operative life.

If compared with previous works, the current activity showed that both AoA and AoS need a complete set of input vector otherwise there is a lack of performances. This is mainly due to complexity of flight dynamics involved in the Piaggio flight simulator. The following input vectors are hence implemented:

(5) $$\begin{array}{cc} \Delta \alpha \; & =f_{\mathrm{VS}-\mathrm{AoA}}\left(TAS,\hat{\alpha },n_{x},n_{y},n_{z},\theta ,\varphi ,p,q,r,\delta_{e},\delta_{a},\delta_{r},\delta_{th},\Delta_{th},\delta_{hs}\right) \end{array}$$

(6) $$\begin{array}{cc} \Delta \beta \; & =f_{\mathrm{VS}-\mathrm{AoS}}\left(TAS,\hat{\alpha },n_{x},n_{y},n_{z},\theta ,\varphi ,p,q,r,\delta_{e},\delta_{a},\delta_{r},\delta_{th},\Delta_{th},\delta_{hs}\right) \end{array}$$

(7) $$\begin{array}{cc} {\left[\Delta \alpha ,\Delta \beta \right]}^{T} & ={\bar{f}}_{\mathrm{VS}-A\&S}\left(TAS,\hat{\alpha },n_{x},n_{y},n_{z},\theta ,\varphi ,p,q,r,\delta_{e},\delta_{a},\delta_{r},\delta_{th},\Delta_{th},\delta_{hs}\right) \end{array}$$

where TAS is the true airspeed, $n_{x}$, $n_{y}$, $n_{z}$ are the accelerations measured by the accelerometers respectively in $X_{B}$, $Y_{B}$ and $Z_{B}$ axes, ψ, θ, ϕ are the Euler angles, *p*, *q*, *r* are the body angular rates, $\hat{\alpha }$ is the initial estimation for the AoA.

For the preliminary design, the synthetic sensors have been tested only with simulated data. The virtual sensors are compared in terms of measurement uncertainty required to COTS or MFP probes from Table 2. However, the Smart-ADAHRS technology has been already compared with vanes in [20] without providing any particular evidence of degradation of the ADS performance. However, this analysis will be conducted in future dedicated experiments. Previous research activities on simulated turbulent environment in [33] showed the possibility of considering previous time steps of the input vector in a time delay network. This practice however is not considered at this stage and will be considered as a further improvement step.

### 3.2. Certification Consideration

As far as certification is concerned, the MIDAS system can be split in three topics: (i) external probes; (ii) electronic unit; (iii) virtual sensors for AoA and AoS.

The external probes, already certified by the supplier, satisfy the required regulations, i.e., TSO-C16A [34] for the Pitot-static probe and the AS793 [35] for the TAT probe.

Design, manufacturing and verification of the electronic unit is one of the main topic of the MIDAS project. In fact, aim of the MIDAS project is to achieve an equivalent DAL-B design assurance level that could be extended to DAL-A for future industrialization. Since all the analytical algorithms are integrated in the field programmable gate array (FPGA), the whole design and validation process will follow RTCA DO-254 [18] guidelines for product assurance and certification. The RTCA DO-178 is not applicable for the MIDAS project. The environmental features will be tested according to requirements established by the RTCA DO-160. In addition to DO-254 risk reduction, pre-qualified (by manufacturer) avionic components will reduce the DO-160 effort and risks as they already integrate lighting protections and other features.

As far as the MIDAS’s virtual sensors for AoA and AoS is concerned, they will be treated as physical sensors and, therefore, they have to satisfy the applicable aeronautical standards. As before mentioned in the Section 1, performance requirements for AoA and AoS usually derive from other systems’ specifications. For example, for autonomous navigation purposes AoA and AoS may be required by the control laws with defined uncertainty in order to achieve desired navigation performance. Therefore, flight mechanics will specify some requirements on the accuracy of AoA and AoS. For the MIDAS project, the AoA and AoS specification are defined by the project leader, Piaggio Aerospace, and published in a project deliverable [36].

Aiming to provide only necessary details for this work, the most significant target performance are summarised in Table 2 where, with a little abuse of notation, for 2σ is intended the value such that the probability $\mathrm{Pr}\left(-2\sigma \;\leq X\leq 2\sigma \;\right)=95.4\%$ also in case the error is not normally distributed. Values reported in Table 2 come from project leader’s system specifications for LFE and EFE.

During the normal and emergency flight conditions, the performance required for AoA and AoS are split into limited flight envelope, LFE, extended flight envelope, EFE, and steady-state flight conditions, SSFC. This latter was proposed by the authors and is more stringent because of the common lack of performance during steady operations [37].

## 4. Reference System

**Figure 4:**
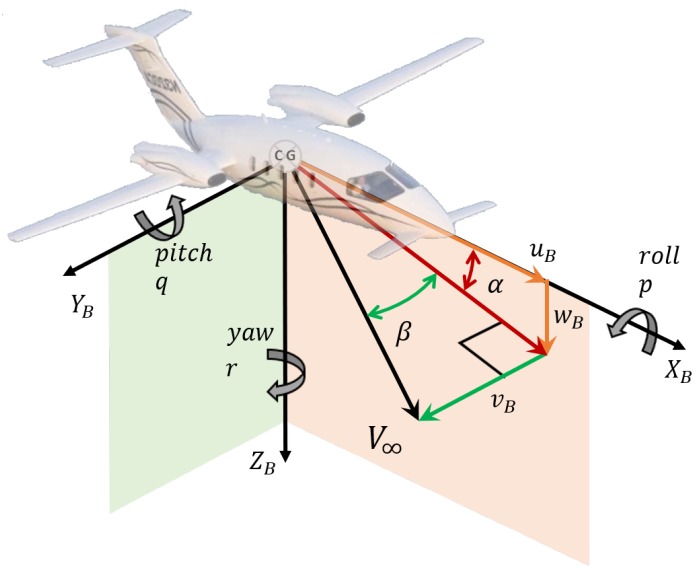
Body reference system.

The present work is developed under regulations declared and published with the Grant Agreement number 821,140 of Clean Sky 2. Data used in this paper are provided by Piaggio Aerospace and they are based on the SAT aircraft model inspired to the Piaggio P180 Avanti aircraft. Therefore, all data used to produce this work are property of Piaggio Aerospace. In favour of the reader, some public information are reported here about the reference aircraft in order to understand better the content of the present work. The reference aircraft has a canard-wing-tail configuration with two pushing propellers. Figure 4 shows the body reference system $\left\{\mathrm{CG},\;X_{B},\;Y_{B},\;Z_{B}\right\}$, the true airspeed vector, $V_{\infty }$, positive directions of attitude angles (roll, pitch, yaw), body angular rates (*p*,*q*,*r*), body linear velocities ($u_{B}$,$v_{B}$,$w_{B}$) and aerodynamic angles, AoA ($\alpha \;=\arctan\frac{w_{B}}{u_{B}}$) and AoS ($\beta \;=\arcsin\frac{v_{B}}{V_{\infty }}$).

In this present work, the aerodynamic angle estimators are fed with surface deflections and throttle. Because of the presence of autopilot modes and control laws, as described in Figure 3, the VS cannot be based on pilot reference commands. Control surfaces and throttle considered for the present work are:

- horizontal canard incidence, $\delta_{hs}$;
- elevator deflection, $\delta_{e}$;
- aileron deflection, $\delta_{a}$;
- rudder deflection, $\delta_{r}$;
- differential throttle, $\Delta_{th}=throttle_{left}-throttle_{right}$;
- mean throttle, $\delta_{th}$.

## 5. Training Strategy

The VS for AoA and AoS will be trained with consolidated strategy based on A/C flying characteristics. Firstly, a set of manoeuvres is defined in order to cover the most of the flight envelope. Once the operative speed range is defined, it is split in several flight regimes, e.g., according to the Mach number. Each flight regime is characterized in terms of its trim conditions: initial velocity, or Mach number, and attitude (usually uniform horizontal path, with null bank and sideslip angle). In each one of the flight regime, the following manoeuvres are simulated:

Each manoeuvre is performed with the aim to excite all A/C dynamic modes. Therefore, the single manoeuvre is repeated several times in order to populate the training pattern with adequate information. For example, the pitch hold is repeated from the minimum to the maximum values with steps of 10 ${}^{\circ }$.

**Figure 5:**
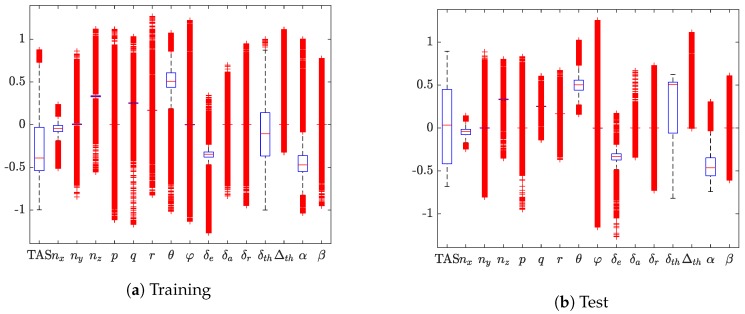
Virtual sensor’s hypercube definition.

Once all data are collected, all variables contained in the input vectors (Equation (7)) are normalized between ±1 considering minimum and maximum values defined by the A/C manufacturer (and not the actual value flown). In this work these values are not shared because of intellectual property reasons. This aspect is crucial for a successful training stage because it will provide a feedback on the correctness of the flown manoeuvres. On the contrary, other flight data will be required to cover the entire region where the VS are defined. As an example, Figure 5 shows how the input variables and the outputs are distributed within the operative range provided by the A/C integrator.

For each single box, the central mark indicates the median and the bottom and top edges of the box indicate the 25th and 75th percentiles, respectively. The whiskers (dashed vertical lines) cover to the most extreme data points not considered outliers whereas the outliers are plotted using the + symbol. The distribution shown in Figure 5 demonstrates that test data collected during simulated flights are included in the training pattern.

## 6. Result

The AoA and AoS estimations rely on the input data provided by the A/C FCS (e.g., inertial data, primary and secondary control surface deflections). In this section results related to flight manoeuvres FT#1–4 are presented because they give a real feedback on the VS performances. The training results are only used for training purposes, e.g., to select the best training among several ones according to consolidated metrics, as showed in previous works [20] and not reported in this paper. Results will be presented as time histories of the errors whereas the current values are not shown for intellectual property reasons. Moreover, some important parameters are derived from result analysis that can be used as pass/fail criteria if compared with those in Table 2.

**Figure 6:**
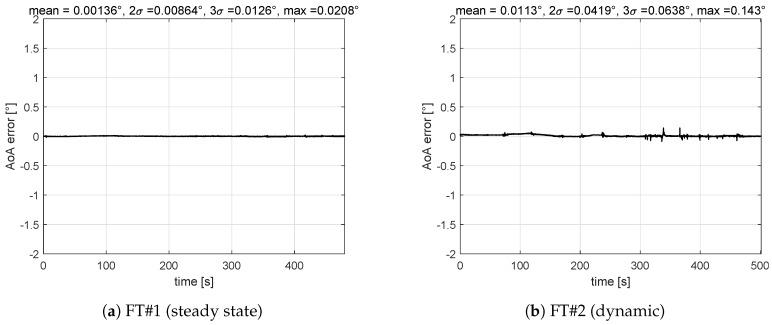

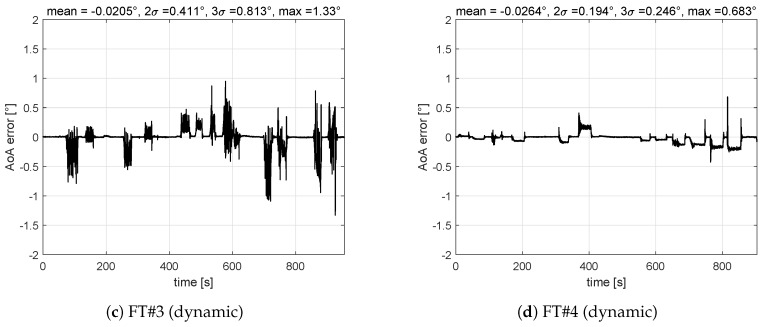
Virtual, analytical or synthetic sensor (VS)-AoA validation results: maximum 2σ (dynamic) = 0.41${}^{\circ }$, maximum error (steady state) = 0.021${}^{\circ }$.

Figure 6 shows time histories of errors obtained with the VS-AoA on the four flight tests and corresponding error distribution. Results are within the required performance (Table 2).

**Figure 7:**
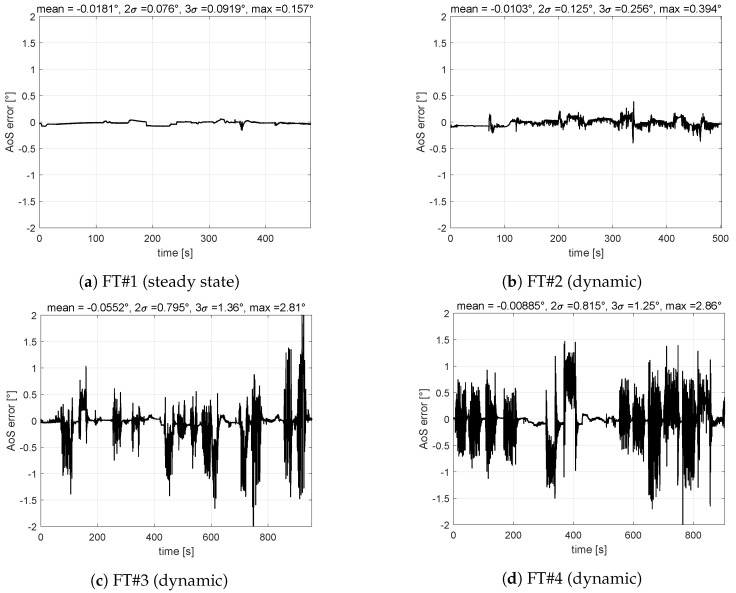
VS-AoS validation results—maximum 2σ (dynamic) = 0.82${}^{\circ }$, maximum error (steady state) = 0.16${}^{\circ }$.

Figure 7 shows time histories of errors obtained with the VS-AoS on the four flight tests and corresponding error distribution. Even though errors of AoS estimation are larger than AoA, they are within the required performance (Table 2).

**Figure 8:**
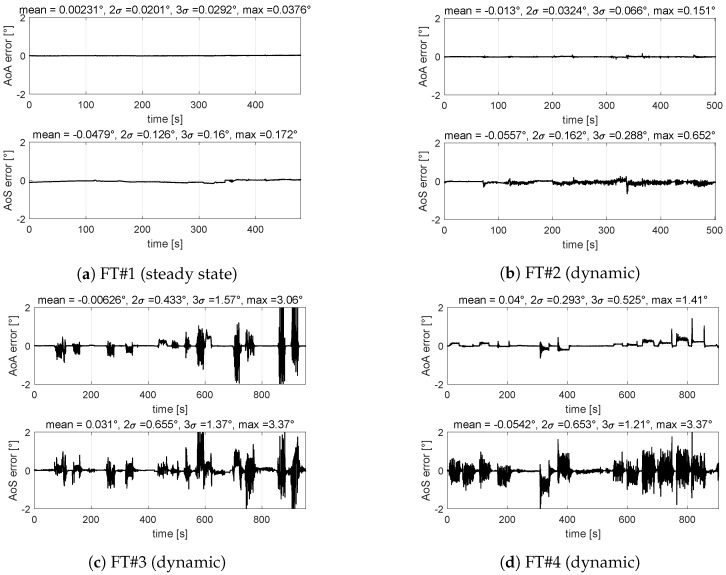
VS-A&S validation results − AoA maximum 2σ (dynamic) = 0.43${}^{\circ }$, maximum error (steady state) = 0.038${}^{\circ }$ − AoS maximum 2σ error (dynamic) = 0.66${}^{\circ }$, maximum error (steady state) = 0.17${}^{\circ }$.

Figure 8 shows time histories of errors obtained with the VS-A&S on the four flight tests and corresponding error distribution. Performances of the double-output neural network are comparable with the two single neural networks (one dedicated to AoA and one to AoS) and, therefore, performance are still acceptable (Table 2).

Results shown in this section allow authors to select the VS-A&S as the candidate virtual sensor architecture because more suitable for real time operations and, more important, for certification aspects (a single software module to be qualified). Moreover, it is confirmed that the AoS estimation shows larger errors with respect to AoA estimation. To understand the reason behind, it was noted that training AoA and AoS at the same time requires a better balanced training pattern between longitudinal flight test points and lateral-directional ones. In fact, AoS was noted to be less than 1${}^{\circ }$ for about 85% of the common training pattern. Therefore, as suggestion for the final design step, a review of the training strategy is required for the MIDAS objectives.

Table 3 collects all validation results obtained with the preliminary design about three aerodynamic angle estimators suitable for civil certification and compared with the most stringent requirements (LFE) provided by Piaggio Aerospace (project leader).

## 7. Conclusions

The present work introduces preliminary design activities for a reliable and ready-to-be-flown air data system for FBW applications within the MIDAS project funded in the frame of Clean Sky 2. The MIDAS technology will enable to remove flow angles vanes or complex rotating/slotted probes that are hard to be procured on the market. The ambition is providing the SAT community with a digital and fully integrated ADS that joins all benefits of available flyable architectures and removes their drawbacks. It is introduced MIDAS projects’ aims to improve the current state-of-the-art of ADS.

It is shown that the MIDAS ADS will be based on AoA and AoS analytical estimators based on neural network techniques, today at TRL6.

Training and test manoeuvres are introduced. Training manoeuvres are defined aiming to excite all dynamic modes of the A/C model whose complexity is increased by several autopilot modes and control laws. Test manoeuvres are collected with the scope to validate results independently from the training set and all possible autopilot configuration.

With respect to the previous works, it emerged that both AoA and AoS need a complete set of input pattern to show acceptable performances due to high complexity of A/C dynamics. According to previous works, AoA and AoS are estimated with dedicated virtual sensors using a single output neural network. This approach has two main drawbacks when applied to qualifiable avionics: (i) it requires a higher computational cost; (ii) two independent software modules to submit to a certification process (DO-178 or, as for the MIDAS project, DO-254). These two main drawbacks suggest to have a single neural network with double output, therefore the computational and certification effort can be drastically reduced.

The virtual sensor (VS-A&S) exhibits only slightly degraded performance for steady-state conditions whereas comparable errors for dynamic flight tests. This evidence makes the single VS (both AoA and AoS) the candidate solution in the next staged of development. Moreover, the A/C complexity conjugated with the new neural network architecture has introduced a new challenge: a common training pattern (both for AoA and AoS) will require, not only, a uniform distribution but even a balanced data between longitudinal and lateral-directional flight test points. This means that the hypercube definition of the neural network shall be uniformly populated as much as possible when collecting flight test data at the simulator. This topic will be discussed with the project leader in a next stage. The reliability analysis and the comparison of the MIDAS technology with classical solution will be studied with the other partners contributions in a next stage.

In conclusion, the selected VS (VS-A&S) exhibits good preliminary performances both for AoA and AoS and it is selected for the candidate VS architecture. Further investigation of larger errors on AoS estimation shall be investigated in the following works.

## Figures and Tables

**Table 1:** Required manoeuvres for training and test data collection and their scope.

Manoeuvre	Scope
Steady flight conditions subgroup 1	train
Steady flight conditions subgroup 2 (FT#1)	test
Sawtooth Glide subgroup 1	train
Sawtooth Glide subgroup 2 (FT#2)	test
Stall – Slow down	train
Pitch Hold	train
Pitch Sweep	train
Bank Hold subgroup 1	train
Bank Hold subgroup 2 (FT#3)	test
Bank Sweep	train
Flat Turn subgroup 1	train
Flat Turn subgroup 2 (FT#4)	test
Steady Heading and Sideslip	train
Dutch roll	train

**Table 2:** High level performance requirements for the AoA and AoS in a limited area of the flight envelope, LFE, in the extended flight envelope, EFE, and in steady-state flight conditions, SSFC.

Data	2σ Error in LFE	2σ Error in EFE	Maximum Error in SSFC
α	0.75 ${}^{\circ }$	1.5 ${}^{\circ }$	0.5 ${}^{\circ }$
β	1.5 ${}^{\circ }$	2.5 ${}^{\circ }$	0.5 ${}^{\circ }$

**Table 3:** Result comparison for the three virtual sensor architectures considered in this work for AoA and AoS estimation.

VS	Data	2σ (Dynamic)	Maximum (Steady State)
VS-AoA	AoA	0.41${}^{\circ }$ < 0.75${}^{\circ }$	0.021${}^{\circ }$ < 0.5${}^{\circ }$
VS-AoS	AoS	0.82${}^{\circ }$ < 1.5${}^{\circ }$	0.16${}^{\circ }$ < 0.5${}^{\circ }$
VS-A&S	AoA AoS	0.43${}^{\circ }$ < 0.75${}^{\circ }$ 0.66${}^{\circ }$ < 1.5${}^{\circ }$	0.038${}^{\circ }$ < 0.5${}^{\circ }$ 0.17${}^{\circ }$ < 0.5${}^{\circ }$

## Abbreviations

The following abbreviations are used in this manuscript:

A/C	Aircraft
ADAHRS	Air data system, attitude and heading reference system
AHRS	Attitude and heading reference system
ADC	Air data computer
ADM	Air data module
ADP	Air data probe
ADS	Air data system
ADU	Air data unit
AoA	Angle-of-attack
AoS	Angle-of-sideslip
CAS	Calibrated airspeed
CG	Center of gravity
COTS	Commercial off the shelf
CS	Certification specifications
DAL	Design assurance level
FBW	Fly-by-wire
FCS	Flight control system
FHA	Failure hazard analysis
FPGA	Field programmable gate array
FT	Flight test
GNSS	Global Navigation Satellite System
LRU	Line replaceable unit
MFP	Multi-function probe
MLP	Multilayer perceptron
OAT	Outside air temperature
SAT	Small air transport
TAS	True airspeed
TAT	Total air temperature
UAM	Urban air mobility
UAS	Unmanned aircraft system
UAT	Universal approximation theorem
VS	Virtual, analytical or synthetic sensor
